# Reading prosocial content in books and adolescents’ prosocial behavior: A moderated mediation model with evidence from China

**DOI:** 10.3389/fpsyg.2022.973481

**Published:** 2022-09-15

**Authors:** Wu Li, Liuning Zhou, Pengya Ai, Ga Ryeung Kim

**Affiliations:** ^1^School of Media and Communication, Shanghai Jiao Tong University, Shanghai, China; ^2^Annenberg School for Communication and Journalism, University of Southern California, Los Angeles, CA, United States; ^3^Wee Kim Wee School of Communication and Information, Nanyang Technological University, Singapore, Singapore

**Keywords:** prosocial behavior, adolescents, reading, prosocial media, moral identity

## Abstract

Drawing upon the General Learning Model, the present study developed a moderated mediation model to provide an in-depth understanding of whether and how adolescents’ reading prosocial content in books predicts their prosocial behavior. The target population in this study is Chinese adolescents, and we adopted a paper-based survey to collect data (*N* = 602). The age range of the sample was from 12 to 19 (*M* = 15.198, *SD* = 1.596). Among all participants, 49.3% were female, and 50.7% were male. PROCESS SPSS Macro was used to analyze the proposed moderated mediation model. The results showed that prosocial content reading was positively associated with adolescents’ prosocial behavior. The positive association included a direct relationship and an indirect relationship through the mediation of moral identity. Furthermore, this study revealed the moderation effect of age on the relationships among prosocial content reading, moral identity, and prosocial behavior. Specifically, as age increases, the effects of prosocial content reading on moral identity and prosocial behavior attenuate, and the mediation effect of moral identity also decreases. The study adds to the body of knowledge on the prosocial media effect by extending it to book reading.

## Introduction

Prosocial behavior represents a broad category of acts that are defined by some significant segments of society and/or one’s social groups as generally beneficial to other people, which include but are not limited to helping others, donating, volunteering, and cooperation (Penner et al., 2005). Prosocial behavior is important to humans because it not only benefits the recipients, but also benefits the actors themselves. For instance, performing prosocial behavior has proved to be effective in improving social relationships and increasing happiness (Aknin et al., 2012; Dunn et al., 2014; Son and Padilla-Walker, 2020). Therefore, research endeavor has been devoted to discovering factors stimulating prosocial behavior in order to better nurture and advance such behavior (e.g., Eisenberg, 2003; Carlo et al., 2010; Imuta et al., 2016).

Scholars from various fields explore the predictors of prosocial behavior from different angles. Psychologists, behavioral economists, and biologists often focus on factors internal to individuals, such as altruistic motivations and prosocial emotions, whereas sociologists typically emphasize social forces external to individuals, including norms, and social networks (Simpson and Willer, 2015). Communication scholars, on the other hand, investigate the impact of the media environment on individuals’ development of prosocial behavior. In the past several decades, they have examined the effects of prosocial content exposure on prosocial behavior focusing on different media, such as television (de Leeuw et al., 2015; Padilla-Walker et al., 2015), movies (de Leeuw and van der Laan, 2018), video games (Gentile et al., 2009; Greitemeyer and Osswald, 2010; World Health Organization, n.d.; Saleem et al., 2012; Prot et al., 2013; Greitemeyer and Mügge, 2014), and music (Greitemeyer, 2009; Ruth, 2016).

Books, on the other hand, are different from the above media types, which can be an interesting case for studying the effect of prosocial content. First, books, constituted by written language, are cultural objects carrying deeply rooted social values (Chen et al., 2020) and generally contain more prosocial content due to the stricter gatekeeping in almost all countries (Xiao and Yu, 2010). Therefore, individuals are more likely to be exposed to prosocial content in reading. Second, compared to watching television and movies, reading books is a more private activity requiring dedication over a longer time, and although people might spend the same amount of time reading a book versus watching a television program, the emotional effect of the former lasts much longer than the latter (Mar et al., 2011). More importantly, according to a Chinese national survey on reading in 2021, the reading rates among adolescents are higher than all other age groups: the reading rate is 99.1% for those 9–13 years old, and 90.1% for those 14–17 years old (National Press and Publication Administration, 2022). Such high reading rates highlight the significance of exploring the effects of reading prosocial content among adolescents.

There has been limited published literature on the prosocial effect of book reading. Mar et al. (2006, 2009) and Mar and Oatley (2008) conducted a series of important studies. With participants recruited from Canada, they explored the link between reading fiction and empathy, as well as the impact of fiction reading on social competency and prosociality. Conducting experiments in the United States, Johnson (2012) and Johnson et al. (2013) empirically demonstrated the influence of reading fiction on empathy, emotional perception and prosocial behavior. They found that being transported into narrative stories could increase empathy and in turn, encourage prosocial behavior (Johnson, 2012). Besides, enhancing imagery in books increases the positive psychological effects of reading stories (Johnson et al., 2013). In addition, a study published in SCIENCE (Kidd and Castano, 2013) conducted five experiments among American participants and the results showed that reading literary fiction temporarily enhances individuals’ theory of mind, the capacity to identify and understand others’ subjective states.

However, there exist some pitfalls which need to be further addressed. First, most studies examine the general activity of fiction reading and lack specificity in terms of reading materials; one specific area to be further explored is prosocial content. Second, besides the frequently examined construct of empathy, some other potential mediators linking reading and prosociality such as moral identity remain underexplored. Moreover, previous studies on the prosocial media content’s effects fail to pinpoint the condition upon which exposure to media content exerts impact on the outcome variables. Finally, most extant studies have been conducted in the context of Western countries, with fairly little attention to Asian countries. Cultural context is especially important for the association between reading and prosocial behavior. There are two reasons. On the one hand, publishing regulation differs from country to country (Li et al., 2019). One the other hand, prosocial behavior highly depends on cultural context because what is prosocial is not understood uniformly across different cultural contexts (Feygina and Henry, 2015). Besides, cultural factors such as interdependence, religion, and social norms are factors influencing prosocial behavior (Feygina and Henry, 2015; Kislyakov et al., 2020). Both country and cultural characteristics need scholarly attention.

To fill the research gaps, we intend to explore whether and how prosocial content reading enhances prosocial behavior among adolescents. We adopt the General Learning Model as our theoretical framework, aiming to extend it to a reading context. We choose adolescents as the interested group in the current study and the reasons for focusing on this certain group are twofold. First, adolescence is the key stage for the advancement of their prosociality (Li et al., 2022), which has proved to be positively associated with their academic performance (Gerbino et al., 2018), friendship quality (Closson, 2009; Jin et al., 2022), wellbeing (Son and Padilla-Walker, 2020), and achievement at later life stages (Toumbourou, 2016). Therefore, to better improve their prosocial behavior, more research endeavors are needed to deepen our understanding of prosociality development among adolescents. Second, adolescents are in a crucial developmental period of worldview exploration marked by instability and uncertainty. They are thus easily susceptible to external environments and more likely to be influenced by media use (Gross, 2004). In addition, to gain a nuanced understanding of the differences among adolescents at different developmental stages, we also investigate the role of age in moderating the impact of prosocial content reading on subsequent prosocial behavior *via* moral identity.

## Literature review

### General learning model

Derived from the General Aggression Model (GAM), which is an integrative social-cognitive model explaining the influence of playing violent video games on people’s antisocial behavior, the General Learning Model (GLM) was developed to explain the formation of people’s more general behavior including nonviolent and prosocial behavior (Bushman and Anderson, 2002; Buckley and Anderson, 2006). According to the GLM, a person’s behavior can be influenced by two kinds of input variables: situational variables and personal variables (Buckley and Anderson, 2006). The former refers to “the features of the environment around the individual” (Buckley and Anderson, 2006, p. 369) such as media and environmental settings, whereas the latter is “what a person brings to the current situation” (Buckley and Anderson, 2006, p. 369) like previous experience and emotions (Buckley and Anderson, 2006). Those input variables affect subsequent reactions or behavior by influencing people’s internal state, including cognition, affect, and arousal (Buckley and Anderson, 2006).

The GLM has been adopted to explain the effects of prosocial video games, in terms of accessibility of prosocial thoughts (Greitemeyer and Osswald, 2011), promotion of prosocial behavior (Gentile et al., 2009), and reduction of aggressive cognitions and behavior (Greitemeyer and Osswald, 2009; Greitemeyer et al., 2012). Its validity has lent itself to the study of other media types such as music (Greitemeyer, 2009) and television (Padilla-Walker et al., 2015). Hence, it will be valuable to extend the GLM to the context of book consumption to test its vitality and validity, which remains underexplored compared to other types of media.

Specific to our study, the situational input variable is adolescents’ exposure to prosocial content in books, measured by their frequency of prosocial content reading, and the personal input variable is adolescents’ age. The internal state influenced by the input variables is moral identity, which is a cognitive construct. The outcome variable is adolescents’ prosocial behavior, the potential behavioral response resulting from the change in moral identity.

### Prosocial content reading and prosocial behavior

Based on the GLM, media as a situational input variable could influence people’s behavior, although the nature of the relationship depends on the media content people are exposed to Greitemeyer and Osswald (2010). In such exposure, individuals will make their own behavioral decisions according to their observation of models’ actions. The positive relationship between exposure to prosocial content and prosocial behavior has been confirmed by studies on prosocial content in television (Mares and Woodard, 2005; de Leeuw et al., 2015), music (Greitemeyer, 2009; Ruth, 2016), and video games (Greitemeyer and Osswald, 2009, 2010; Saleem et al., 2012; Greitemeyer and Mügge, 2014).

Specific to the prosocial effect of book reading, the literature is limited with mixed results. For instance, Johnson (2012) demonstrated the positive influence of fiction reading on individuals’ empathy, emotional perception, and prosocial behavior. A meta-analysis revealed that compared to nonfiction reading and no reading, fiction reading leads to small yet statistically significant improvement in social-cognitive performance (Dodell-Feder and Tamir, 2018). Yet, research by Małecki et al. (2018), which contains three studies investigating the effects of fiction reading on pro-animal attitudes and behavior, found a positive impact in one study while a negative impact in the other two. One main reason for such discrepancy, as we see it, is the absence of a focus on prosocial content reading in the research design.

As discussed earlier, the GLM maintains that people could “learn” prosocial behavior *via* observing media characters’ prosocial behavior. Although there has been no empirical support that exposure to prosocial book content facilitates prosocial behavior, the positive relationship confirmed in the study of other media provides theoretical foundation for our study. Therefore, we propose the first hypothesis as follows:

**H1:** Prosocial content reading positively predicts prosocial behavior.

### Moral identity

Moral identity, referred to as “the degree to which a person identifies himself or herself as a moral person” (Zhu et al., 2011, p. 151), is crucial in people’s moral functioning due to its influence on people’s interpretations and responses in situations involving moral judgments and decisions (Shao et al., 2008; Hardy and Carlo, 2011). Currently, the social-cognitive perspective dominates the literature in defining moral identity (Gotowiec and van Mastrigt, 2019), and it views moral identity as “a self-conception organized around a set of moral traits” (Aquino and Reed, 2002, p. 1427). Also, Aquino and Reed (2002) recognize the dual dimensionality of moral identity as composed of internalization and symbolization. Internalization is the private aspect of moral identity, which reflects “the degree to which the moral traits are central to the self-concept” (Aquino and Reed, 2002, p. 1427), while symbolization refers to the public aspect of moral identity, which reflects “the degree to which the traits are reflected in the respondent’s actions in the world” (Aquino and Reed, 2002, p. 1427). The internalization dimension is more reliable in predicting moral outcomes than the symbolization dimension (Boegershausen et al., 2015), so this study adopts the internalization dimension of moral identity in its operationalization.

From the social-cognitive perspective, like other social identities that make up a person’s social self-schema, an individual’s moral identity can be activated (Aquino et al., 2009) or suppressed by contextual, situational, or even individual-differences variables (Aquino and Reed, 2002; Forehand et al., 2002), though the mechanisms of its formation are still relatively ambiguous (Hardy and Carlo, 2011). Researchers have examined the factors influencing individuals’ moral identity. For instance, Aquino et al. (2009) found that situational factors such as reading Ten Commandment, financial incentives, and writing with moral laden terms can activate the current accessibility of moral identity and in turn affect the sequential moral behavior intentions. Zhu et al. (2011) argued that the symbolic modeling of leaders could influence their followers’ moral judgment. Furthermore, the review by Hardy and Carlo (2011) showed that several factors, including developmental contexts (e.g., religious involvement and parenting style), individual characteristics (e.g., academic achievement), and opportunities to behave morally (e.g., community service), contribute to the development of moral identity. However, little is known about the influence of media use on moral identity.

On the other hand, moral identity as an internal state may mediate the relationship between input and outcome variables, according to the GLM (Buckley and Anderson, 2006). To the best of our knowledge, no research has directly addressed the relationship between reading prosocial content and moral identity; however, studies on prosocial media use could provide valuable theoretical guidance to explore a potential relationship. For example, the accessibility of prosocial thoughts is found to function as a pathway linking playing video games and prosocial behavior (Greitemeyer and Osswald, 2010), and such mediation effect is also found in listening to songs with prosocial lyrics (Greitemeyer, 2009). Considering that moral identity is similar to prosocial thought accessibility as both can be seen as how easy the schema can be primed or accessed (Greitemeyer and Osswald, 2010), we propose that moral identity could also be learned and activated when individuals are exposed to prosocial content in books. In other words, prosocial content reading positively influences moral identity.

Meanwhile, moral identity has been described as a self-regulatory mechanism that motivates moral actions and has also consistently been found to be a significant antecedent to prosocial behavior (Reynolds and Ceranic, 2007; Hertz and Krettenauer, 2016; Ding et al., 2018; Gotowiec and van Mastrigt, 2019). A meta-analysis conducted by Hertz and Krettenauer (2016) revealed that moral identity is significantly related to moral behavior, including prosocial behavior. Based on the social-cognitive perspective, the accessibility of one’s moral identity determines the possibility of action (Shao et al., 2008), and a stronger moral identity denotes a more central role for morality in a person’s identity. Therefore, a person’s moral self-schema has a higher probability of being activated, and he or she is more likely to engage in moral behavior (Aquino and Reed, 2002; Aquino et al., 2009; Hardy and Carlo, 2011). Furthermore, people would make efforts to sustain the consistency between their moral self-conception and their behavior: as people possess a stronger moral identity, they will try to behave morally to maintain such consistency (Aquino and Reed, 2002; Aquino et al., 2009; Hardy and Carlo, 2011). As such, it is safe to say that moral identity positively predicts prosocial behavior. In sum, in light of the above discussions, we develop the second hypothesis.

**H2:** Prosocial content reading positively predicts moral identity, which in turn positively predicts prosocial behavior.

### Age

Different components of moral development, such as moral judgment, moral reasoning, theory of mind, and antisocial behavior, are all correlated with age (McDonald and Stuart-Hamilton, 1996; Happé et al., 1998; Forney et al., 2005; Heiphetz and Liane, 2014). Krettenauer and Victor (2017) discuss moral identity as a developmental construct that grows with age, and Kingsford et al. (2018) highlight the significance of developmental issues in moral identity emergence. Yet, the association between moral identity and age is not always a linear relationship. For instance, it was found that external moral identity motivation decreased with age, whereas internal moral identity motivation increased with it, and the effects of age were stronger in adolescence and emerging adulthood than in young adulthood and middle age (Krettenauer and Victor, 2017). In one word, there are no definitive answers to the relationship between age and moral identity.

Similarly, research into the association between age and prosocial behavior is not conclusive. Eisenberg et al. (2005) point out that despite theoretical assumptions that are consistent with an increase in prosocial tendencies in adolescence and early adulthood, empirical results are mixed. Different aspects of prosocial functioning, such as perspective-taking, prosocial moral reasoning, and simple prosocial proclivities (e.g., helping and sympathy), are associated with age in different and not necessarily linear ways. Jirsaraie et al. (2019) also indicate an inconsistency in the relationship between age and prosocial behavior, and age is found to be strongly related to cooperation and helping attitudes but fails to predict charitability.

Based on the notion of the GLM, personal factors could interact with situational factors such as reading prosocial content (Buckley and Anderson, 2006). Therefore, age, a personal factor, could influence the association between reading prosocial content, moral identity, and prosocial behavior. Similarly, Valkenburg and Peter (2013) suggested that media effects are not uniform across individuals but are made complicated by potential moderators. One of them is developmental susceptibility, an individual’s responsiveness to media as a result of his or her cognitive, emotional, and social development (Valkenburg and Peter, 2013, 2017; Valkenburg, 2020). This argument highlights the salience of incorporating age into media effects studies (Cingel et al., 2020; Valkenburg, 2020). Although current literature does not offer a definitive answer to whether age could moderate such a relationship and what the interaction effect would be, theories on media effects are essential to understanding when or under which conditions prosocial content reading impacts prosocial behavior. For this study, we believe that age (a personal variable) interacts with prosocial content reading (a situational variable) to exert an interaction effect on moral identity and prosocial behavior. Previous research has suggested that younger people are more susceptible to prosocial influences (Foulkes et al., 2018). Therefore, we propose the following hypotheses:

**H3a**: Age moderates the relationship between prosocial content reading and moral identity. The relationship attenuates as age increases.

**H3b**: Age moderates the relationship between prosocial content reading and prosocial behavior. The relationship attenuates as age increases.

**H3c**: Age moderates the mediation effect of moral identity on the association between prosocial content reading and prosocial behavior. The indirect relationship attenuates as age increases.

**FIGURE 1:**
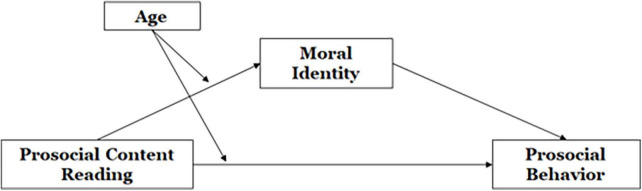
Hypothesized model.

Based on the above hypotheses, we proposed our conceptual model as depicted in Figure 1.

## Materials and methods

### Participants and procedures

The target population of this study is adolescents. According to the definition of the World Health Organization, adolescence refers to the group aged from 10 to 19 years (World Health Organization, n.d.). We employed survey method to collect data to test the proposed model. The study was approved by the Institutional Review Board at the authors’ institution (No. H2021177I). The research procedures are described below.

We adapted established scales measuring the key constructs of our study, and then consulted three experienced middle school teachers with regards to the scales’ wording to make sure that they were fully comprehensible to adolescent students. Then we performed a pretest with a convenience sample of 103 adolescents to test the reliability and validity of all the instruments. Necessary modifications were made based on the pretest results, including dropping several items and improving the wording of the remaining ones.

We conducted the formal survey among middle and high school students in a mid-sized city located in East China over 3 weeks in January 2021. We randomly selected one middle school and one high school from the city and invited several teachers to distribute the paper-based questionnaire during breaks. Participants were randomly selected from each grade at each school to ensure the distribution of our participants covered all six grades. The number of participants from each grade was approximately one hundred.

A total of 647 students participated in the study. We removed from the sample participants who filled in the survey with almost identical responses, whose questionnaires were almost blank, and whose responses were contradictory (especially with respect to reversed items). After this procedure, a final sample of 602 was kept for data analysis.

### Measures

To test the proposed model and the hypothesized relationships in our study, the instruments must have both reliability and validity. We first conducted confirmatory factor analysis (CFA) and dropped three items whose loading value was smaller than 0.6 (Comrey and Lee, 2013). The results from another CFA regarding the remaining items showed that all the items’ standardized factor loadings were greater than 0.6 and the model fit indices of the measured model were acceptable (χ^2^/*df* = 2.931;*CFI* = 0.943;*TLI* = 0.934;*RMSEA* = 0.057, 90%*CI* = [0.050, 0.063];*SRMR* = 0.037). As displayed in Table 1, the Cronbach’s alpha of all constructs ranges from 0.814 to 0.887, surpassing the acceptable level of 0.70 (Nunnally, 1978). The composite reliability (CR) of these measures ranges from 0.815 to 0.903, meeting the acceptable level of 0.60 (Fornell and Larcker, 1981). These indicators indicate that our measurement items have a high degree of internal reliability. The average variance extracted (AVE) of prosocial behavior is 0.438 (those of the other two constructs are 0.597 and 0.622), which is below the recommended level of 0.50. Nevertheless, according to Fornell and Larcker (1981), AVE is a relatively conservative estimate to assess the validity of the measurement, and “on the basis of ρ_η_ (composite reliability) alone, the research may conclude that convergent validity of the construct is adequate, even though more than 50% of the variance is due to error” (p. 46). Their recommendation is also followed by later researchers in their studies (e.g., Lam, 2012; Pervan et al., 2018). Therefore, we conclude that the convergent validity of the instruments in our study is acceptable considering the CR of each construct is well above the recommended level. Meanwhile, as shown in Table 1, all the square roots of the AVE values were higher than the correlation between the variable and other variables, indicating good discriminant validity (Fornell and Larcker, 1981). These variables were operationalized as follows.

**TABLE 1:** Descriptive analysis, correlational coefficients, reliability, and validity test.

	*M*	*SD*	Cronbach’s α	CR	AVE	1	2	3
(1) Prosocial content reading	2.710	1.011	0.814	0.815	0.597	0.772		
(2) Prosocial behavior	3.598	0.758	0.887	0.903	0.438	0.296***	0.662	
(3) Moral identity	4.141	0.844	0.827	0.830	0.622	0.143***	0.471***	0.789

***p < 0.001. Diagonal SQRT of AVE.

#### Prosocial content reading

The scales for prosocial content reading in our study were adapted from the prosocial media use scale of Ostrov et al. (2006) and Prot et al. (2013). The definition of prosocial behavior was provided for participants first. We also provided the illustration of prosocial book content, which refers to any book content encouraging prosocial behavior, such as description of people doing good deeds, stories of moral exemplars, and content encouraging harmonious interpersonal relationships. Participants were first instructed to list three of their favorite books, and for each book participants were then asked to rate how frequently they encountered prosocial content on a 5-point scale (1 = never and 5 = extremely frequently). The score for prosocial content reading was calculated as the average of the three items.

#### Prosocial behavior

Based on the study of Carlo and Randall (2002), Yang et al. (2016) developed the Chinese version of the prosocial behavior scale incorporating features of Chinese adolescents which has been validated and extensively used in measuring adolescents’ prosocial behavior in China. Thus, we adopted it for the current study. Participants were asked to self-report the frequency of engaging in such behavior as described in the items on a 5-point scale (1 = Never and 5 = Always). Sample items included “I voluntarily give seats to those in need, such as the elderly, the weak, the sick, the disabled, and the pregnant,” “When a classmate is sick, I take him/her to see the school nurse,” and “I take the initiative to say ‘Hi’ to new classmates and make friends with them.”

#### Moral identity

The measurement of moral identity was adapted from Aquino and Reed (2002) and Wan and Yang (2008). A list of words *(trustworthy, honest, filial, responsible, sincere, polite, kind, loyal, upright*, and *helpful*) describing personal traits was provided to participants who were asked to visualize in their mind the kind of person who matched these characteristics. Participants were then asked to imagine how that person would think, feel, and act. After participants had a clear image of what this person would be like, they were instructed to rate the degree to which they agree or disagree with related statements on a 5-point Likert scale (1 = Strongly Disagree and 5 = Strongly Agree). The statements included “It would make me feel good to be a person who has these characteristics,” “Being someone who has these characteristics is an important part of who I am,” and “I strongly desire to have these characteristics.”

## Data analysis and results

### Descriptive statistics

Data analyses were conducted on the valid sample of 602, and missing values were interpolated by the mean value. The age range of the sample was from 12 to 19 (*M* = 15.198, *SD* = 1.596). Among all participants, 49.3% were female, and 50.7% were male. Gender is included as a control variable because it is repeatedly found to be associated with both moral orientation (Jaffee and Hyde, 2000) and prosocial behavior (Pursell et al., 2008). The descriptive statistics of the variables in our study and correlational coefficients between each pair of them are also listed in Table 1.

### Mediation effect analysis

We performed the mediator analysis using Model 4 in PROCESS SPSS Macro (Hayes and Andrew, 2012) to examine the mediation effect from prosocial content reading to prosocial behavior *via* moral identity. Age and gender were entered into the model as covariates. As Table 2 indicates, at 0.05 statistical significance level prosocial content reading positively correlates with moral identity (*B* = 0.111, *p* < 0.001) and prosocial behavior (*B* = 0.170, *p* < 0.001). Moral identity positively correlates with prosocial behavior (*B* = 0.386, *p* < 0.001).

**TABLE 2:** Regression analysis results.

Dependent variable	Moral identity				Prosocial behavior			
		
	Coefficient	*SE*	95% *CI*		Coefficient	*SE*	95% *CI*	
						
			*LL*	*UL*			*LL*	*UL*
**Independent variable**								
Prosocial content reading	0.111**	0.033	0.045	0.176	0.170***	0.026	0.119	0.221
Moral identity					0.386***	0.032	0.324	0.448
Age	0.007	0.021	–0.035	0.049	−0.053**	0.016	–0.085	–0.021
Gender (male = 1)	−0.251***	0.068	–0.384	–0.118	–0.096	0.053	–0.200	0.008
Constant	3.863***	0.342	3.191	4.536	2.391***	0.292	1.818	2.963
**Model fit**								
*R*	0.207				0.539			
*R* ^2^	0.043				0.291			
*F*	8.931***				61.187***			

***p < 0.001, **p < 0.01.

Considering that using the *p*-value of the regression model to judge mediation effects is not solid enough (Hayes and Rockwood, 2017), the bias-corrected bootstrapping method was also used to examine the mediation effects of moral identity. A 95% bias-corrected confidence interval based on 5,000 bootstrap samples indicated that the indirect effect through moral identity does not straddle zero (*B* = 0.043, 95% CI = [0.016, 0.073]), and at the same time the direct effect of prosocial content reading on prosocial behavior does not straddle zero (*B* = 0.170, 95% CI = [0.119, 0.221]). Therefore, moral identity partially mediates the relationship between prosocial content reading and prosocial behavior.

### Moderated mediation effect analysis

Model 8 of PROCESS SPSS Macro (Hayes and Andrew, 2012) was later used to examine the conditional mediation model. As shown in Table 3, after the interaction term of prosocial content reading and age was added, prosocial content reading was again found to be positively related with moral identity (*B* = 0.775, *p* = 0.017) and prosocial behavior (*B* = 0.784, *p* = 0.002). Moral identity is also positively related with prosocial behavior (*B* = 0.379, *p* < 0.001). The interaction term of prosocial content reading and age is negatively related with moral identity (*B* = −0.044, *p* = 0.040) and prosocial behavior (*B* = −0.041, *p* = 0.015).

**TABLE 3:** Regression analysis results.

Dependent variable	Moral identity				Prosocial behavior			
		
	Coefficient	*SE*	95% *CI*		Coefficient	*SE*	95% *CI*	
						
			*LL*	*UL*			*LL*	*UL*
**Independent variable**								
Prosocial content reading	0.775*	0.325	0.137	1.412	0.784**	0.252	0.289	1.279
Moral identity					0.379***	0.032	0.317	0.442
Age	0.132*	0.065	0.006	0.259	0.063	0.050	–0.035	0.161
Interaction term^a^	−0.044*	0.021	–0.086	–0.002	−0.041*	0.017	–0.073	–0.008
Gender (male = 1)	−0.246***	0.068	–0.379	–0.113	–0.093	0.053	–0.196	0.011
Constant	1.959*	0.987	0.022	3.897	0.657	0.765	–0.846	2.159
**Model fit**								
*R*	0.223				0.546			
*R* ^2^	0.050				0.298			
*F*	7.792***				50.562***			

^a^Interaction term = prosocial content reading × age; ***p < 0.001, **p < 0.01, *p < 0.05.

**FIGURE 2:**
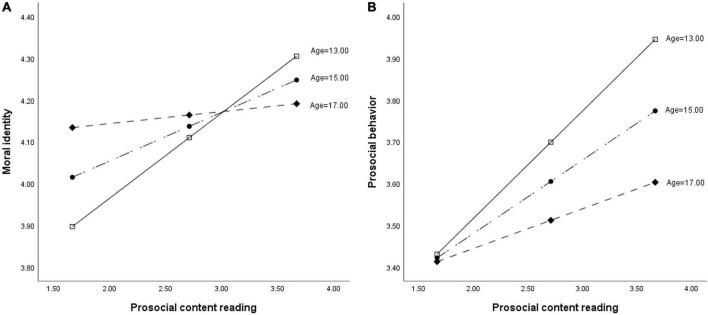
The moderation effect of age.

To better illustrate the interaction effect of age and prosocial content reading on moral identity and prosocial behavior, we plotted the interaction effects in Figures 2A,B. As displayed in both figures, the slopes are the largest among early adolescents, while lowest in late adolescents. In other words, the positive effects of prosocial content reading on moral identity and prosocial behavior decrease as age increases.

Again, the bias-corrected bootstrapping method was employed to examine the moderated mediation effects of moral identity. As Table 4 shows, the direct effect of prosocial content reading on prosocial behavior exists at all age levels (the bootstrapping confidence intervals do not contain zero). Taking into consideration the significant effect of the interaction term in ordinary least squares regression (OLS) Model 4, we determined age moderates the direct effect of prosocial content reading on prosocial behavior. Specifically, although the effect sizes of the direct effects are significant at all age levels, they become weaker as age increases.

**TABLE 4:** The conditional direct and indirect effect.

	Direct effect				Indirect effect			
		
Age	Effect	*SE*	*LLCI*	*ULCI*	Effect	*BootSE*	*BootLLCI*	*BootULCI*
13.000	0.257	0.044	0.171	0.344	0.077	0.026	0.031	0.133
15.000	0.176	0.026	0.125	0.227	0.044	0.015	0.018	0.075
17.000	0.095	0.040	0.016	0.174	0.011	0.018	−0.025	0.047

Indirect effects are based on 5,000 bootstrap samples. LLCI, lower limit confidence interval; ULCI, upper limit confidence interval.

Meanwhile, the index of moderated mediation of age is −0.017 (*BootSE* = 0.009, 95% *CI* = [−0.034, −0.001]), indicating a significant moderating effect of age on the indirect relationship between prosocial content reading and prosocial behavior through moral identity. Taking a closer look at Table 4, we found that the indirect effect *via* moral identity exists among early and middle adolescents (the bootstrapping confidence intervals do not contain zero), and the effect size is stronger for early adolescents. In addition, there is no indirect effect in the group of late adolescents (the bootstrapping confidence interval contains zero). In short, as age increases, the indirect effect of adolescents’ prosocial content reading on prosocial behavior through moral identity attenuates and even disappears.

## Discussion

Drawing upon the GLM, this study examined the relationship between prosocial content reading and adolescents’ prosocial behavior. All hypotheses were confirmed, with the results supporting the effectiveness of the GLM in the new context of prosocial content reading. The theoretical implications of our research are fourfold.

First, this study goes beyond fiction and non-fiction reading to examine the positive effect of prosocial content reading on prosocial behavior among adolescents. Young (2019) suggested that reading can cultivate virtue in three ways: enhancing empathy, self-reflection, and social learning. While the general reasoning for the positive effects of reading fictions is that experiencing simulated social world benefits the development of social skills (Mar et al., 2009), this study looks into the effects of reading from the social learning perspective. This finding is consistent with the claim of the GLM in that exposure to the media content makes people “learn” how to behave. In other words, when reading content encouraging prosocial behavior, such as stories depicting protagonists who help others, adolescents will “learn” that such behavior is desirable and behave the same through vicarious learning.

Second, this study introduces moral identity as an alternative mediator to other constructs like empathy, helping us better understand the underlying mechanism of how book reading affects prosocial behavior. Previous studies on the effects of prosocial media content or fiction reading usually adopts empathy as the mediator (Johnson, 2012; Prot et al., 2013). However, in spite of being one of the most important antecedents to prosocial behavior, it is not as consistent and internalized as moral identity. Our finding of the partial mediation effect of moral identity suggests that with exposure to prosocial content in books, adolescents will not only take prosocial actions by just mimicking the depicted prosocial behavior, but also through the change of their moral identity, which means they will internalize the concept that they should behave morally.

More importantly, this study discovers that the direct and indirect effects of prosocial content reading on prosocial behavior are moderated by adolescents’ age. Though previous studies on prosocial behavior have suggested age as an important factor (Cingel et al., 2020), little is known about its moderating effect on the relationship between prosocial media use and prosocial behavior. This study reveals that as the age of adolescents increases, the impacts of prosocial content reading on both moral identity and prosocial behavior decrease, and the size of the mediating effect of moral identity decreases as well. These findings shed light on the boundaries of the effects of prosocial media content. This is in line with the notion of the Differential Susceptibility to Media Effects Model, which suggests that more attention should be paid to the heterogeneity of media effects (Valkenburg and Peter, 2013). Particularly, this study shows that the prosocial media effects can be different across different stages of adolescence. Based on this finding, future media effect studies should put more emphasis on the heterogeneity of groups in a more nuanced way.

Finally, the present study enriches our understanding of the prosocial media effect by extending it to book reading. Drawing on the GLM, our study identifies the impact of reading prosocial content in books on adolescents’ prosocial behavior and uncovers the underlying mechanism as well. It represents one important attempt to explore the prosocial effect of book reading from a media use perspective, and our findings add to the body of knowledge on the prosocial effect of media use. Previous studies have suggested prosocial behavior varies across different media types, while the association between book reading and prosocial behavior is stronger than other media types (Li et al., 2019). In future research, it would be interesting to explore why there are such differences. This study also extended the GLM to the context of book reading. Previous research has tested the GLM on video games (Gentile et al., 2009; Greitemeyer and Osswald, 2011), music (Greitemeyer, 2009) and television (Padilla-Walker et al., 2015). To the best of our knowledge, this is the first study to examine the relationship between reading books and prosocial behavior based on the GLM.

Apart from theoretical contributions, the current study has important practical implications. For instance, our results shed light on the positive role of prosocial content reading in promoting adolescents’ prosocial behavior; therefore, it is valuable for those who develop or administer adolescent reading practice or programs, such as school teachers, librarians, and parents, to encourage adolescents to engage in more prosocial content reading. Besides, as the mediating role of moral identity is only significant among younger adolescents, the abovementioned parties should nurture adolescents’ habit of prosocial reading as early as possible in their developmental stage in order to effectively improve their moral identity and ensuing prosocial behavior. Since the findings supported the potential of utilizing books to encourage prosocial behavior, this study also provides valuable lessons for publishers’ business practices. Publishers can publish books containing more prosocial content and recommend them to teachers and parents. According to the moderation effect, publishers can label the books as including more prosocial content for younger adolescents so as to reap more educational and commercial benefits.

This study is not without limitations. First, the data of this study is cross-sectional; thus, we should exercise caution in the causal inference of the results. Future studies can use longitudinal or experimental design to investigate possible causal relationships among prosocial content reading, moral identity, and prosocial behavior in adolescents. Second, the survey was conducted in a mid-sized city in East China, which limits the generalizability of our research findings. The same investigation could be conducted in more cities in China as well as in other countries to improve the external validity of the results. Furthermore, we must acknowledge this study is flawed with social desirability problem in research design because of the self-reported measures. In the future, field experiment with actual prosocial behavior, such as donation, can be conducted to reduce the influence of social desirability.

## Data availability statement

The raw data supporting the conclusions of this article will be made available by the authors, without undue reservation.

## Ethics statement

The studies involving human participants were reviewed and approved by Institutional Review Board of Shanghai Jiao Tong University. Written informed consent to participate in this study was provided by the participants’ legal guardian/next of kin.

## Author contributions

WL: conceptualization, methodology, reviewing, and editing. LZ: conceptualization, reviewing, and editing. PA: methodology, data collection, data analysis, and writing. GK: data analysis. All authors contributed to the article and approved the submitted version.
